# Hepatitis C Virus Diversification in Argentina: Comparative Analysis between the Large City of Buenos Aires and the Small Rural Town of O'Brien

**DOI:** 10.1371/journal.pone.0084007

**Published:** 2013-12-30

**Authors:** Marcelo D. Golemba, Andrés C. A. Culasso, Federico G. Villamil, Patricia Bare, Adrián Gadano, Ezequiel Ridruejo, Alfredo Martinez, Federico A. Di Lello, Rodolfo H. Campos

**Affiliations:** 1 Cátedra de Virología, Facultad de Farmacia y Bioquímica, Universidad de Buenos Aires, Buenos Aires, Argentina; 2 Unidad de Trasplante Hepático, Hospital Británico de Buenos Aires, Argentina y Unidad de Trasplante Hepático, Hospital El Cruce, Buenos Aires, Argentina; 3 Sección Virología, Academia Nacional de Medicina, Buenos Aires, Argentina; 4 Hepatology Unit, Hospital Italiano de Buenos Aires, Buenos Aires, Argentina; 5 Centro de Educación Médica e Investigaciones Clínicas Norberto Quirno, CEMIC, Buenos Aires, Argentina; University of Cincinnati College of Medicine, United States of America

## Abstract

**Background:**

The estimated prevalence of HCV infection in Argentina is around 2%. However, higher rates of infection have been described in population studies of small urban and rural communities. The aim of this work was to compare the origin and diversification of HCV-1b in samples from two different epidemiological scenarios: Buenos Aires, a large cosmopolitan city, and O'Brien, a small rural town with a high prevalence of HCV infection.

**Patients and Methods:**

The E1/E2 and NS5B regions of the viral genome from 83 patients infected with HCV-1b were sequenced. Phylogenetic analysis and Bayesian Coalescent methods were used to study the origin and diversification of HCV-1b in both patient populations.

**Results:**

Samples from Buenos Aires showed a polyphyletic behavior with a t_MRCA_ around 1887–1900 and a time of spread of infection approximately 60 years ago. In contrast, samples from ÓBrien showed a monophyletic behavior with a t_MRCA_ around 1950–1960 and a time of spread of infection more recent than in Buenos Aires, around 20–30 years ago.

**Conclusion:**

Phylogenetic and coalescence analysis revealed a different behavior in the epidemiological histories of Buenos Aires and ÓBrien. HCV infection in Buenos Aires shows a polyphyletic behavior and an exponential growth in two phases, whereas that in O'Brien shows a monophyletic cluster and an exponential growth in one single step with a more recent t_MRCA_. The polyphyletic origin and the probability of encountering susceptible individuals in a large cosmopolitan city like Buenos Aires are in agreement with a longer period of expansion. In contrast, in less populated areas such as O'Brien, the chances of HCV transmission are strongly restricted. Furthermore, the monophyletic character and the most recent time of emergence suggest that different HCV-1b ancestors (variants) that were in expansion in Buenos Aires had the opportunity to colonize and expand in O’Brien.

## Introduction

Hepatitis C virus (HCV) infection is a major cause of chronic hepatitis, liver cirrhosis and hepatocellular carcinoma [1], [2]. About 150 million people worldwide are chronically infected with HCV, and more than 350 000 people die every year from HCV-related liver diseases. Taxonomically, HCV is classified into six major genotypes (1 to 6) and into many subtypes based on phylogenetic analysis [3], [4]. Genotype distribution differs by epidemiological characteristics and geographical areas [5]. Before the adoption of anti-HCV control measures in blood banks (∼1990), HCV was mainly transmitted via blood transfusion. Today, needle sharing among injecting drug users is the most common form of HCV transmission [6], [7]. Therefore, the distribution of certain subtypes such as HCV-1a, 1b and 3a is largely related to risk factors such as blood transfusions, intravenous drug use or inadequately sterilized medical equipment [5], [8], [9].

The prevalence of HCV infection varies among different geographic regions. Kershenobich et al. has recently published a review describing the status of HCV infection in six countries of Latin America, including Argentina [10]. These authors observed that HCV prevalence ranges from 1.0 to 2.3% and that genotype 1 is the most prevalent in the countries analyzed [10].

In Argentina, the available information is limited and heterogeneous. Although a Consensus Conference estimated an overall HCV prevalence of around 2% (Consenso Argentino de Hepatitis C, 2007), marked differences have been noted along the country. The highest reported prevalence has been observed in population-based studies performed in small urban and rural towns [11]–[15]. More precisely, a recent study in the small rural town of Wheelwright (Santa Fe, Argentina), which has a 4.9% prevalence of infection, revealed a monophyletic clade and a time for the most recent common ancestor (t_MRCA_) dated around 1950. The study showed that HCV infection was more frequent in people older than 50 years, with the highest prevalence (22%) in the group aged between 70–79 years [16]. A similar situation has been reported from ÓBrien, another small rural town of approximately 2300 inhabitants located in Buenos Aires Province [14]. The overall prevalence of HCV infection in O’Brien was found to be 5.6%, 0.6% in individuals aged <40 years and 12.6% in those >40 years, with a peak of 23% in the group aged 60–70 years. All patients from O’Brien were infected with HCV genotype 1b. However, the information about the origin of HCV infection was evaluated from the epidemiological forms and phylogenetic analysis was restricted. Therefore, it would be interesting to perform a strong phylogenetic analysis and complement it with a coalescence analysis in order to compare the diversity and origin of samples from small towns and a large cosmopolitan city.

Evolutionary analyses of virus sequences are now an important tool in molecular epidemiology. These methods have been previously used to evaluate the relationships between viral gene sequences and/or to estimate epidemiological history [16]–[26]. Therefore, the aim of this study was to compare the origin and diversification of HCV-1b in two populations of Argentina with different epidemiological characteristics, by means of phylogenetic and Bayesian coalescent analysis.

## Materials and Methods

### Design and Study Population

From June 1996 to July 2010, the patients infected with HCV genotype 1b who received their first therapy against HCV infection were prospectively followed at four tertiary care centers from Buenos Aires (a large city with 13 million inhabitants). A basal serum sample was collected from each patient and stored at −80°C for subsequent determinations. A total of 46 individuals from this cohort were randomly selected from the four hospitals: CEMIC (n = 10), Italian Hospital (n = 21), Lanari Hospital (n = 8) and Muñiz Hospital (n = 7). On the other hand, 37 samples randomly selected from 93 patients infected with HCV genotype 1b from O’Brien, a small rural town in Buenos Aires province, epidemiologically described by Picchio et al. [14], were also included in this study. Written informed consent to participate in this study was obtained from all patients of the participating hospitals. The protocol of this study was approved by the ethics committee of the Facultad de Farmacia y Bioquímica of the Universidad de Buenos Aires (Exp #701283).

### RNA Extraction, cDNA Synthesis and DNA Amplification

RNA was extracted from 150 ml serum samples using a commercial reagent (Trizol, Invitrogen) following the manufacturer’s instructions. Reverse transcription reactions and the E1/E2 (672 nt, positions 1416–2087) and NS5B (486 nt, positions 8139–8624) genes were amplified by a hemi-nested PCR and the amplicons sequenced as described previously [16]. The D90208 (HCV-J) was used as reference sequence.

### Phylogenetic Analysis

Phylogenetic analysis was performed using the E1/E2 and NS5B genomic regions by separate and concatenated datasets from Buenos Aires and O’Brien (except from samples from Lanari and Muñiz Hospitals, where only NS5B was analyzed due to lack of serum availability). On the other hand, 352 HCV-1b sequences from GenBank were added as reference [including 55 sequences from the Wheelwright outbreak previously described in [16]]. Additionally, two sequences of HCV-1a and two of HCV-1c were included as out-group. Thus, the total population included in the phylogenetic analysis consisted of 439 sequences. These sequences were aligned with ClustalX v2.0.10 program [27].

Phylogenetic trees were constructed using distance Neighbor-Joining, Maximum Likelihood (ML) and Parsimony methods. Evolutionary models were inferred according to the Akaike Information Criterion (AIC) statistics obtained with the jModelTest v0.1.1 program [28]. The distance analysis was performed with the PAUP* v4.0b10 program [29]. The ML analysis was performed with the RAxML v7.2.8 program [30] and the parsimony analysis was performed with the TNT v1.1 program [31], [32]. The robustness of the reconstructed phylogenies was evaluated by bootstrap analysis (1000 replica), and the phylogenetic trees were analyzed using the Dendroscope v2.7.4 program [33].

### Divergence Time and Population Dynamics

Bayesian coalescent-based methods were used to estimate the t_MRCA_ and the population dynamics in: a) Buenos Aires dataset and b) O’Brien dataset (n = 36, without sample O529, which did not belong to O’Brien cluster). This analysis was performed for the E1/E2 and NS5B regions and the concatenated matrix of both regions. The estimates of the t_MRCA_ and the population dynamics were obtained by means of the Bayesian Markov Chain Monte Carlo techniques implemented in the BEAST v1.6.1 program [34]. Both strict and relaxed uncorrelated lognormal molecular clock were enforced. The demographic histories of Buenos Aires and O’Brien datasets were reconstructed using a flexible model known as Bayesian Skyline Plot (BSP) implemented in the BEAST v1.6.1 program [34], [35].

As the O’Brien datasets were isochronous and the Buenos Aires datasets presented a weak temporal structure assessed by Path-O-Gen v1.4 program, we could not estimate the t_MRCA_ parameter reliably, and so used external information to estimate the t_MRCA_ and population dynamics. For this reason, it was necessary to incorporate an external nucleotide substitution rate as previously described by Magiorkinis et al. [21]. Thus, as prior, the regions were calibrated with a normal distribution. The mean rate of substitution obtained and introduced as a prior for E1/E2 was 2.28×10^−3^ s/s/y (stdev = 4.17×10^−4^), that for NS5B was 6.10×10^−4^ s/s/y (stdev = 1.96×10^−4^) and that for the concatenate analysis was 1.22×10^−3^ s/s/y (stdev = 2.64×10^−3^). For the Buenos Aires dataset, the sampling time was incorporated in the analysis since the inclusion do not affect the time of the t_MRCA_. On the other hand, the t_MRCA_ for the samples from O’Brien was inferred using a normal distribution with a mean value of 50 years and a standard deviation of 20 years as prior distribution, based on our belief about the history of the epidemic.

Finally, the best substitution model inferred according to the AIC statistics obtained with the jModelTest v0.1.1 program [28] was selected for each dataset and implemented in the BEAUti program. The convergence of the parameters to a stationary distribution was assessed with the TRACER v1.5.0 program [36] **,** and the models compared by a Bayes Factor analysis [37].

### Nucleotide Sequence Accession Numbers

GenBank accession numbers for the sequences obtained in this study are KF733833–KF733900 for the E1/E2 sequences and KF733901–KF733983 for the NS5B sequences.

## Results

### Main Characteristics of the Patients Studied

Forty-six patients from Buenos Aires and 37 from O’Brien were analyzed. Twenty-seven (58.7%) patients from Buenos Aires and 19 (51.3%) from O’Brien were male. The median (Interquartile Range) age for Buenos Aires was 58 (50–66) years and for O’Brien 63 (53–68). Among Buenos Aires patients, 44 (95.7%) were HCV-monoinfected and 2 (4.3%) were HIV/HCV-coinfected, whereas all patients from O’Brien were HCV-monoinfected. Risk factors for HCV infection in patients from Buenos Aires were blood transfusions in 10 of them (21.7%), use of intravenous drugs (IDU) in 7 of them (15.2%), and sexual contact in 2 of them (4.3%). The route of infection in the remaining 25 patients (54.3%) was unknown.

All 37 (100%) HCV-positive patients from O’Brien had received injections with non-disposable and inadequately sterilized syringes and needles administered by the same health care professional. In addition, 29 out of the 37 patients (78.4%) had undergone dental care with the same professional who had used non-disposable and re-sterilized materials. Other risk factors included surgical procedures in 25 (67.6%) patients and blood transfusions in 9 (24.3%).

### Phylogenetic Analysis

**Figure 1:**
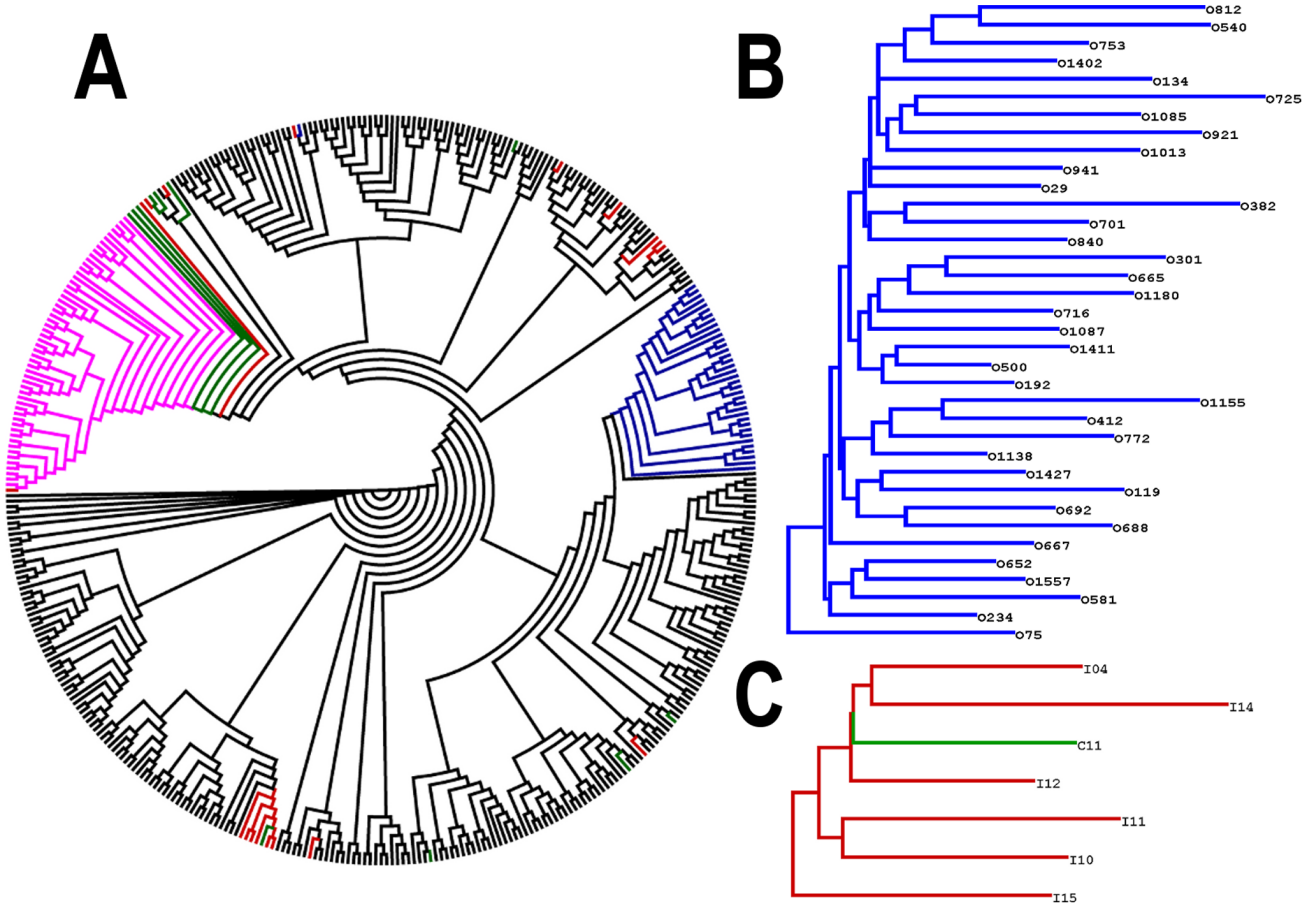
Phylogenetic tree from the concatenated analysis for ML. A) Corresponds to the global analysis of concatenated sequences. B) O’Brien cluster pruned from A (Bootstrap = 100%). C) Cluster BA7 pruned from A (Bootstrap = 98%). Red branches correspond to Italian Hospital, green branches to CEMIC, magenta branches to Wheelwright and black branches to the reference sequences.

In the separate analyses, principally for the NS5B region, the bootstrap values were lower than those in the concatenated analysis. Thus, the increase in the bootstrap values for the concatenated analysis could be the result of lower phylogenetic signal in the datasets from separate analyses (data not shown). For this reason, the phylogenetic analysis of concatenated regions was the one taken into account. This analysis showed that all sequences from O’Brien, except one (O529), formed a single monophyletic cluster. In addition, this cluster was independent of the Wheelwright cluster previously described by our group [16] (Figure 1). In contrast, most samples from Buenos Aires (CEMIC and Italian Hospital) appear intermingled among the reference sequences, showing a polyphyletic behavior (Figure 1). However, seven of them form a small cluster (BA7) that includes one sequence from CEMIC and six sequences from the Italian Hospital (Figure 1).

**Figure 2:**
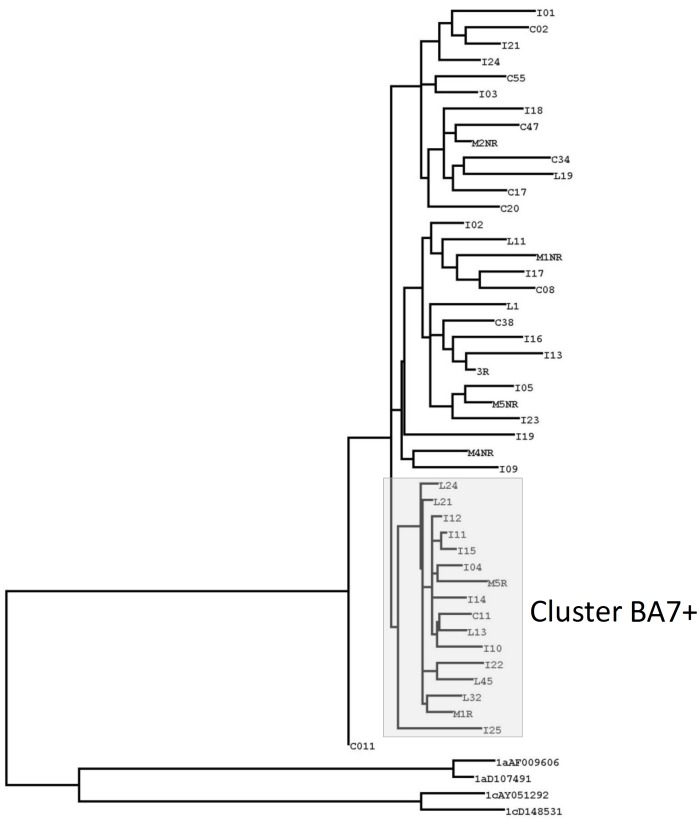
ML Phylogenetic tree from NS5B region. The box shows cluster BA7+ (Bootstrap value = 31%). C, I, L and M corresponds to the sequences from CEMIC, Italian Hospital, Lanari Hospital and Muñiz Hospital, respectively. Two sequences of HCV-1a and two sequences of HCV-1c were used as an out-group.

To extend our phylogenetic study, we performed an analysis for NS5B, which included more samples from Buenos Aires. Here, we observed that cluster BA7 incorporates seven sequences from Lanari and Muñiz Hospitals and was denominated cluster BA7+ (Figure 2). However, the I22 sequence of Italian Hospital was incorporated into this cluster. This sequence was not grouped in cluster BA7 in the phylogenetic analysis shown in figure 1.

### Coalescent Bayesian Analysis

The E1/E2 and NS5B partial sequence genes from the samples from Buenos Aires and O’Brien were used to estimate both the t_MRCA_ and population dynamics. From the Bayes Factor analysis, the lognormal relaxed molecular clock model was the one that best fit the data for E1/E2 and concatenated matrices. Nonetheless, there was no significant difference between the strict and lognormal relaxed molecular clock for the NS5B region. Therefore, for the NS5B region, the strict molecular clock was selected to avoid over-parameterization. Table 1 shows the t_MRCA_ values obtained from the samples from Buenos Aires calculated from the BSP model. Similar results were obtained when analyzing the other demographic models (constant population size, exponential growth, expansion growth, logistic growth) that achieved convergence, suggesting that these results are robust to demographic history (data not shown).

**Table 1:** Estimates of the t_MRCA_ for Buenos Aires and O’Brien samples by Bayesian coalescent analysis under the BSP model.

Datasets	Concatenated	E1/E2	NS5B
	Year (HDP 95%)	Year (HDP 95%)	Year (HPD 95%)
Buenos Aires	NA	NA	1887 (1805–1935)
Buenos Aires (without L and M)	1905 (1838–1933)	1939 (1917–1956)	1900 (1795–1957)
Buenos Aires (without BA7+)	NA	NA	1866 (1784–1933)
BA7+	NA	NA	1959 (1941–1978)
O’Brien	1950 (1934–1964)	1961 (1949–1972)	1961 (1940–1998)

HPD = highest posterior density (95%HPD).

NA = Not available.

**Figure 3:**
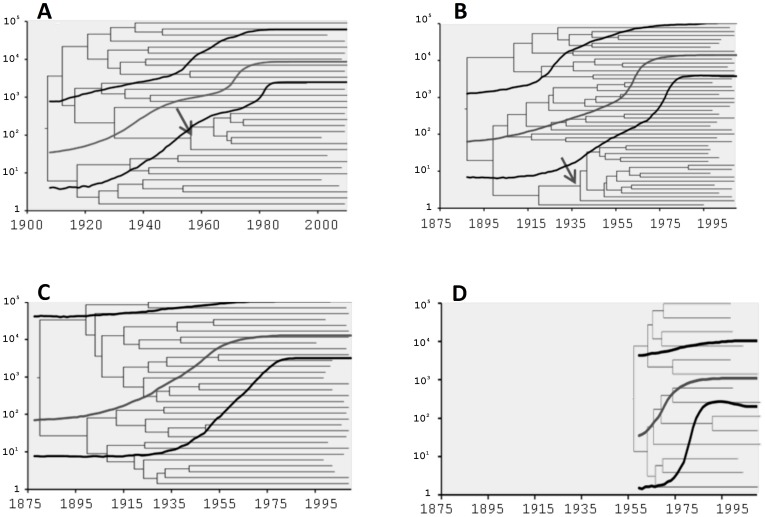
Demographic profiles and coalescence trees obtained by the BSP model. A) CEMIC and Italian Hospital, concatenated sequences. B) Buenos Aires, NS5B sequences. C) Buenos Aires without cluster BA7+. D) BA7+ sequences. The lines in the demographic curves correspond to the median value and the estimated HPD95%. The arrow in A and B represents the node of clusters BA7 and BA7+, respectively. The length of the branches of the tree represents the time (year).

Additionally, the population dynamics for all four Buenos Aires datasets were analyzed through the different demographic profiles obtained with the BSP model (Figure 3). The first dataset showed an exponential growth in the diversity measured as effective number of population multiplied by the generation length (N_e_×τ) from around 1920 until about 1980 and then remained constant until the present (Figure 3A).

Interestingly, the diversification of cluster BA7, whose emergence would be responsible for the second phase in the effective number of population, occurred in this period. Figure 3B shows a similar curve but in this case, the second step could be due to the formation of cluster BA7+, which includes samples from Lanari and Muñiz Hospitals. In figure 3C, without cluster BA7+, a softer curve was observed with only one step of exponential growth. Finally, figure 3D describes the exponential growth of cluster BA7+.

**Figure 4:**
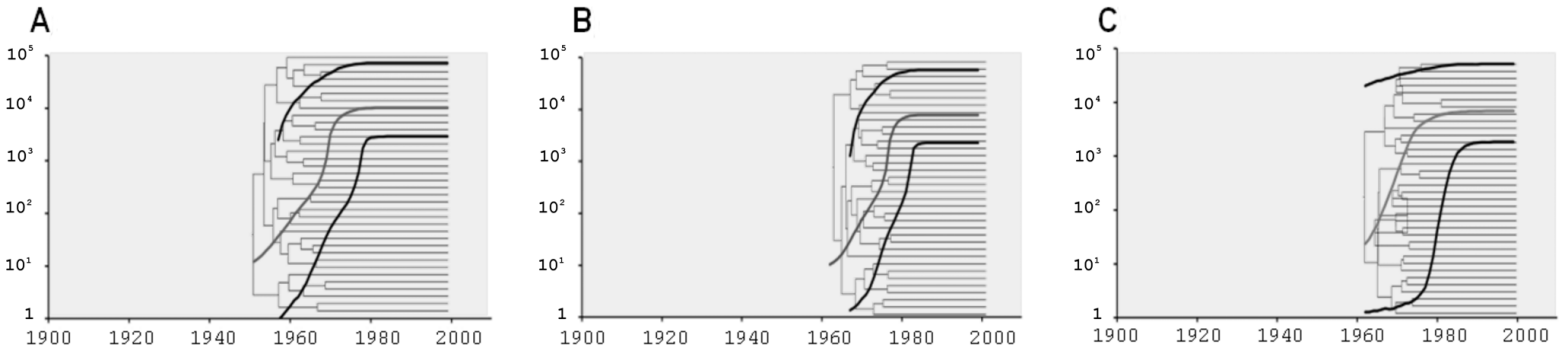
Demographic profiles and coalescence trees obtained by the BSP model for O’Brien sequences. A) Concatenated sequences, B) E1/E2 analysis and C) NS5B analysis. The length of the branches of the tree represents the time (year). The lines in the demographic curves correspond to the median value and the estimated HPD95%. The length of the branches of the tree represents the time (year).

On the other hand, the t_MRCA_ for the samples from O’Brien (HPD: High Posterior Density) was around 1950 (1934–1964) for the concatenated analysis, 1961 (1949–1972) for E1/E2 and 1961 (1940–1998) for NS5B (Table 1). It is important to note that the same results were obtained when the prior for the t_MRCA_ (based on our belief about the history of the epidemic) was not included in the analysis (data not shown). Besides, the demographic profile obtained for O’Brien sequences describes a rapid exponential growth along 15–20 years, which then remains constant until the present (Figure 4).

## Discussion

Demographic differences between cities and small towns are reflected in the molecular epidemiology of viral infections. In this study, analysis of HCV sequences from infected patients from Buenos Aires and O’Brien showed important differences in regard to phylogeny, origin and dispersion. While HCV infection in Buenos Aires had a polyphyletic behavior and an exponential growth in two phases, that in O’Brien showed a monophyletic cluster and an exponential growth in one single step, with a more recent t_MRCA_.

Kershenobich et al. has recently reported an analysis of previous works on the epidemiology of HCV infection from six countries throughout Latin America (Argentina, Brazil, Mexico, Puerto Rico, Peru and Venezuela). These authors found that the estimated HCV prevalence in the continent ranged from 1.0 to 2.3%, with a predominance of genotype 1 infection. However, the authors highlighted the limitations of the available epidemiological data and the need for further research [10].

In the present study samples from HCV-1b-infected patients from a large and cosmopolitan city such as Buenos Aires were analyzed and compared with samples from HCV-1b-infected patients from O’Brien, a small rural town with a high prevalence of HCV-1b infection. Using the E1/E2 and NS5B regions of HCV genome, we observed different patterns in both populations. Phylogenetic analysis performed on the E1/E2 and/or NS5B regions has been widely used in HCV epidemiological studies and is therefore recommended for this kind of analysis [16], [19], [21], [25], [26], [38], [39].

Most Buenos Aires sequences were intermingled with different reference sequences from other countries (polyphyletic origin), supporting the hypothesis of multiple virus introductions possibly related to the immigration processes that occurred during the 20^th^ century in Argentina. A particular situation was observed for a small monophyletic cluster (BA7+), which was detected in samples from all participating institutions. This monophyletic cluster possibly reflects the spread of a particular lineage in Argentina with a similar source of transmission. In contrast, all samples from O'Brien, with only one exception (O529), were grouped into a single monophyletic clade. Sample O529 belonged to a patient who had received multiple blood transfusions in 1974, before the adoption of anti-HCV control measures in blood banks, and who may have therefore not become infected by the same transmission route. In addition, the O’Brien cluster was reciprocally independent of the Wheelwright cluster previously reported [16], although there was a similar epidemiological situation in both towns. These results suggest that both groups of sequences (ÓBrien and Wheelwright) may possibly reflect a transmission of a particular viral lineage, through a common source of infection, but independent in both towns. In fact, epidemiological data based on patient interviews from ÓBrien strongly suggest that the use of unsafe injection practices was the main risk factor for HCV infection [14].

Importantly, in certain analyses, some sequences from Buenos Aires were phylogenetically related to the O’Brien or Wheelwright clusters. For this reason, when assessing a possible outbreak or a single case of transmission, it is important to add the strongest evidence available to the phylogenetic analysis. Therefore, in our study we incorporated the largest number of sequences available from around the world and used several phylogenetic methods.

The t_MRCA_ for Buenos Aires HCV-1b samples was estimated around 1887–1900. As previously mentioned, the most likely explanation for this date is the multiple introductions of HCV-1b in Argentina as a consequence of the immigration from European countries (mainly Spain and Italy) at the beginning of the 20^th^ century. In addition, the t_MRCA_ for Buenos Aires is similar to that calculated for HCV-1b circulating in Brazil, Chile and Spain [19], [26], [40]. On the other hand, the t_MRCA_ for the samples from O’Brien was estimated about 1950–1960 and was similar to that calculated for cluster BA7+ (∼1960) and Wheelwright outbreak (∼1950) [16].

The skyline plot profiles obtained for the samples from Buenos Aires (without considering the samples from Lanari and Muñiz Hospitals) of concatenated sequences showed a two-step curve with an initial period of exponential growth in the N_e_×τ from the t_MRCA_ until ∼1960. After this first period, a second and more pronounced period of growth of about 10 years (1970–1980) was observed. Accordingly, the increase in the growth rate of the N_e_×τ that occurred between 1970 and 1980 coincides with the diversification of cluster BA7. Furthermore, in the demographic profile obtained from the NS5B sequences from Buenos Aires, we observed an increase in the N_e_×τ approximately between 1960 and 1970 that corresponds with the diversification of cluster BA7+. For cluster BA7+, we observed a pronounced and exponential growth in the N_e_×τ from ∼1959 (t_MRCA_) to ∼1975, which then remained constant until the present. These results are consistent with the increase observed in the N_e_×τ between 1960 and 1970 at the end of the curve, and allow us to speculate that the two-step curve is the product of the viral diversification of cluster BA7+, while HCV-1b was already expanding in Buenos Aires. Moreover, we observed that the demographic profile and t_MRCA_ obtained for cluster BA7+ were similar to those obtained for the samples from O'Brien. These demographic profiles of viral spread suggest that there was a high rate of initial transmission followed by a process of low or null transmission. In addition, all the datasets analyzed showed that the exponential growth of HCV-1b reached a plateau around 1980, which is consistent with that previously reported by Magiorkinis et al. [21]. This exponential decrease in the growth was observed prior to the implementations of anti-HCV screening in the early 1990s [41].

A limitation of this study is that the E1/E2 region for some samples from Buenos Aires (Lanari and Muñiz Hospitals) was not sequenced. Nevertheless, this fact does not reduce the interest of the findings reported herein, since the use of NS5B has proven to be useful in the phylogenetic analysis of HCV [19]. Another limitation and important point to mention is that most samples from Buenos Aires and O'Brien analyzed were isochronous. However, this inconvenient was solved through the estimation of the t_MRCA_ and population dynamics by the incorporation of an external substitution rate of the virus (a prior with normal distribution and high standard deviation) and the use of epidemiological data for O’Brien dataset. The use of this external substitution rate possibly caused an error in the HPD95% values estimated in the analyses.

In conclusion, in a large cosmopolitan city like Buenos Aires, the polyphyletic origin and the spread of HCV infection are in agreement with a longer period of expansion, while, in small towns like O’Brien, the chances of transmission are strongly restricted. Furthermore, the monophyletic character and the older time of emergence in O’Brien suggest that different HCV-1b ancestors (viral variants) that were in expansion in Buenos Aires had the opportunity to colonize and expand in O’Brien.
